# Treatment of tumour tissue with radio‐frequency hyperthermia (using antibody‐carrying nanoparticles)

**DOI:** 10.1049/nbt2.12061

**Published:** 2021-07-09

**Authors:** Reza Didarian, Ibrahim Vargel

**Affiliations:** ^1^ Department of Nanotechnology and Nanomedicine Hacettepe University Beytepe Campus Ankara Turkey; ^2^ Department of Plastic Reconstructive and Aesthetic Surgery Hacettepe University, Sıhhiye Ankara Turkey

## Abstract

Intelligent inorganic nanoparticles were designed and produced for use in imaging and annihilating tumour cells by radio‐frequency (RF) hyperthermia. Nanoparticles synthesised to provide RF hyperthermia must have magnetite properties. For this purpose, magnetite nanoparticles were first synthesised by the coprecipitation method (10–15 NM). These superparamagnetic nanoparticles were then covered with gold ions without losing their magnetic properties. In this step, gold ions are reduced around the magnetite nanoparticles. Surface modification of the gold‐coated magnetic nanoparticles was performed in the next step. A self‐assembled monolayer was created using cysteamine (2‐aminoethanethiol) molecules, which have two different end groups (SH and NH_2_). These molecules react with the gold surface by SH groups. The NH_2_ groups give a positive charge to the nanoparticles. After that, a monoclonal antibody (Monoclonal Anti‐N‐CAM Clone NCAM‐OB11) was immobilised by the 1‐ethyl‐3‐(3‐dimethylaminopropyl)carbodiimide/N‐hydroxysuccinimide method. Then, the antenna RF system (144.00015 MHz) was created for RF hyperthermia. The antibody‐nanoparticle binding rate and cytotoxicity tests were followed by in vitro and in vivo experiments. As the main result, antibody‐bound gold‐coated magnetic nanoparticles were successfully connected to tumour cells. After RF hyperthermia, the tumour size decreased owing to apoptosis and necrosis of tumour cells.

## 1 INTRODUCTION

Cancer is one of the most important diseases of humans. It is a group of diseases characterised by the uncontrolled growth and spread of abnormal cells, possibly resulting in death if the spread is not controlled. Although considerable progress has been achieved in the diagnosis and treatment of cancer, current levels are not satisfactory [1, 2, 3].

Cancer is treated with surgery, radiation, chemotherapy, hormones, and immunotherapy. The disease will be most treatable if malignancies are detected before cells become cancerous, or at an early stage [4]. Because early diagnosis is associated with positive results, there are many incentives for developing technologies that can detect cancer at its earliest stages [5, 6, 7].

Complementary techniques in cancer treatment are used to improve the life quality of cancer patients alongside standard methods. However, it is unclear whether these methods are effective, and the effect varies among patients. Accordingly, clinical trials cannot be considered the main method [8].

In the hyperthermia method, the physiological temperature (37°C) of the tumour area is raised a few degrees (∼40–45°C) and thus cells die via apoptosis and necrosis [9, 10]. Different methods are used for hyperthermia energy, one of which is radio‐frequency (RF) hyperthermia, in which hyperthermia is provided by applying waves on magnetite and gold‐coated magnetite nanoparticles [11].

Magnetic nanoparticles (MNPs) are a major class of nanoscale materials with the potential to revolutionise current clinical diagnostic and therapeutic techniques, providing some attractive possibilities in biomedicine. Owing to their unique physical properties and ability to function at the cellular and molecular level of biological interactions, MNPs are actively being investigated as the next generation of magnetic resonance imaging (MRI) contrast agents [12, 13] and as carriers for targeted drug delivery [14]. Although early research in the field dates back several decades, the surge of interest in nanotechnology has significantly expanded the breadth and depth of MNP research [15, 16]. MNPs with a wide range of applications in the detection, diagnosis, and treatment of disease (such as cancer [17, 18], cardiovascular disease [19], and neurological disease [20]) may have a significant role in meeting the health care needs of the future. MNPs can be manipulated by an external magnetic field gradient; hence, they can be made to deliver a package, such as an anticancer drug, to a targeted region of the body, such as a tumour [21, 22].

Gold salt can be reduced at the surface of MNPs to add an Au layer, which can then be functionalised with thiolated molecules via a covalent bond to the surface of MNPs [23].

Gold‐coated MNPs are biocompatible for both human and animal applications and can easily be detected using their optical properties (for various biomedical applications) [24, 25]. Chemical structures of nanoparticles are stable and are nontoxic. The gold‐coating method provides good surfaces for the next biological functionalisation [26]. Gold‐coated MNPs are used in antibody binding, gene transfer, and antisense therapy. When gold‐coated MNPs carry molecules in the body, they increase the stability, bioavailability, and survival of molecules [27, 28].

Given that (almost) any substance may create monoclonal antibodies that specifically bind to that substance, they can serve to detect or purify the substance. Specific receptors exist on tumour cells that can be used to identify them. Monoclonal antibodies connect specifically to the receptor surface on tumour cells [29, 30].

## 2 EXPERIMENTAL SECTION

### 2.1 Synthesis and characterisation of magnetite nanoparticles

The coprecipitation method (mechanical stirrer at high temperature) was used in this synthesis. In the N_2_ atmosphere, 24 ml of 1.25 M Fe^+2^ and Fe^+3^ salt solution and 24 ml of 5 M NaOH solution were added to 32 ml of deionised water at 80°C. The black solution was stirred at 2000 rpm for 2 h. Magnetic particles and nonmagnetic particles were separated with the help of magnetic separators and then washed several times with water. Nanoparticles were stabilised with 0.1 M tetramethylammonium hydroxide solution [31]. Then, nanoparticles were characterised by ZetaPALS, Fourier transform infrared (FTIR), vibrating sample magnetometer (VSM), X‐ray diffraction (XRD), X‐ray photoelectron spectroscopy (XPS), and transmission electron microscopy (TEM) analyses.

### 2.2 Synthesis and characterisation of gold‐coated magnetic nanoparticles

MNPs (1.2 ml) were dispersed in deionised water (100 ml). Then, 0.7 ml of 0.155 M trisodium citrate was added to the solution and sonicated for 15 min. The suspension was heated to boiling (96°C) at a high stirring speed. Gold ions around the MNPs were reduced by adding 10 ml of 0.01 M tetrachloroaurate, and was stirred at boiling point for 15 min. The suspension was centrifuged at 15,000 rpm, and then magnetic and nonmagnetic nanoparticles were separated by a magnet. Oxalate solution dissolved the uncoated MNPs. The suspension was mechanically stirred in oxalate (0.02 M) for 1 h to separate gold‐coated magnetite nanoparticles [32, 33, 34]. Then, nanoparticles were characterised by ZetaPALS, FTIR, VSM, XRD, XPS, and TEM analyses.

### 2.3 Surface modification of gold‐coated magnetic nanoparticles

Cysteamine solution (1 mM) was prepared. Then, the solution was prepared with gold‐coated MNPs (1 ml) and cysteamine (225 μl) and shaken in the dark for 12 h. A magnet was used to separate the nanoparticles from the suspension, followed by the characterisation of nanoparticles with ZetaPALS and FTIR analyses.

### 2.4 Immobilisation of antibody on nanoparticles

Monoclonal antibodies (Monoclonal Anti‐N‐CAM Clone NCAM‐OB11, Sigma‐Aldrich) were immobilised to the synthesised nanoparticles. Cysteamine functionalised‐gold‐coated MNPs were dispersed in the phosphate‐buffered solution (pH 7.4). Antibody and nanoparticle solutions were mixed in the 1:3 (v/v) ratio, and then 1‐ethyl‐3‐(3‐dimethylaminopropyl)carbodiimide (0.2 M) and N‐hydroxysuccinimide (0.05 M) were added to the activating –COOH group present in the antibody. The activated carboxyl group binds to the –NH_2_ group of cysteamine functionalised‐gold‐coated MNPs, leading to formation of the amide bond. The solution was washed with phosphate buffer (pH 7.4) several times and then collected by centrifugation [35]. The nanoparticles were analysed with FTIR and ninhydrin test for characterisation.

### 2.5 Radio‐frequency generator

A handmade RF generator was used. The specific absorption rate (SAR) simulations were studied for frequencies from 64 to 600 MHz by Winter et al. [36]. The SAR index for 144.00015 MHz (applied here) was considered to be 0.281 (plausible value). In these studies, cell lines and nude mice were induced by exposure after treatment with the nanoparticles.

## 3 RESULTS AND DISCUSSION

### 3.1 Size and zeta potential analysis

MNPs were synthesised at different sizes, with an average size of 63 nm. The average zeta potential of the MNPs was –18 mV. The average size and zeta potential of gold‐coated MNPs were 75 nm and −30 mV, respectively; thus, the thickness of the gold‐coating is 12 nm. The variations in size and zeta potential in the MNPs are the reason for the gold coating. The average size and zeta potential of cysteamine functionalised gold‐coated MNPs were 98 nm and −7 mV, respectively. The working principle of the zeta size is based on dynamic light scattering. Because of the limitations of this method, the actual size of nanoparticles was smaller than their graphic size; their dimensions were obtained from TEM analysis (Table 1).

**TABLE 1:** Hydrodynamic size distribution and zeta potential of synthesised nanoparticles

	Size (nm)	Zeta potential (mV)
Magnetic nanoparticle	63	−18
Au at SPIONs	75	−30
Cysteamine functionalised Au at SPIONs	98	−7

Abbreviation: Au at SPIONs, gold‐coated superparamagnetic iron oxide nanoparticles.

### 3.2 Fourier transform infrared analysis

**FIGURE 1:**
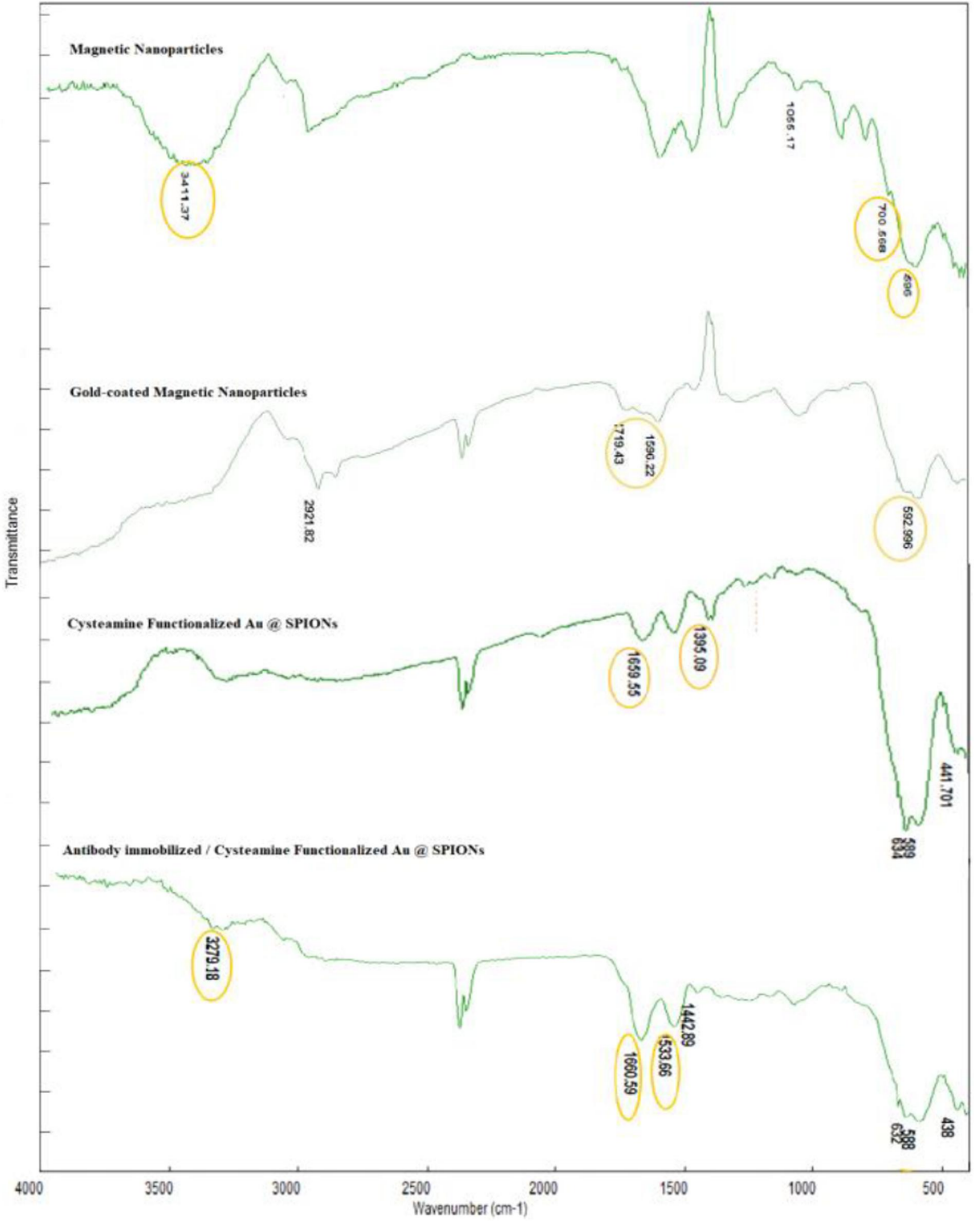
Fourier transform infrared of synthesised nanoparticles

In the MNPs, observed bands at 596 and 700 correspond to the Fe‐O bond vibration of iron oxide nanoparticles. The peak at 3411 is attributed to the stretching vibrations of OH, which belongs to OH absorbed by Fe_3_O_4_ nanoparticles (Figure 1) [37].

In the FTIR spectra of the gold‐coated sample, the peak at 596 shifted to 592 and the peak at 700 completely disappeared. Trisodium citrate was used as a reducing agent to coat the MNPs with gold; hence, the bands observed at 1719 cm^–1^ and 1596 cm^–1^ waves are the trisodium citrate carboxylate group and the peak for CH_2_‐O deformation, respectively. These results indicate the coating of Fe_3_O_4_ particles by gold (Figure 1) [37].

In the FTIR spectra of the cysteamine functioned gold‐coated sample, the bands observed at 1659 and at 1395 correspond to N–H bond vibration and C–N bond vibrations, respectively (Figure 1) [37, 38].

In the FTIR spectra of antibody immobilised gold‐coated MNPs, the peak at 1660 corresponds to C = O bond vibration. The peaks at 3280 and 1534 belong to the N–H bond of the secondary amine and the N–H bonds of the secondary and the primary amides. respectively (Figure 1) [37, 38].

### 3.3 Vibrating sample magnetometer analysis

**FIGURE 2:**
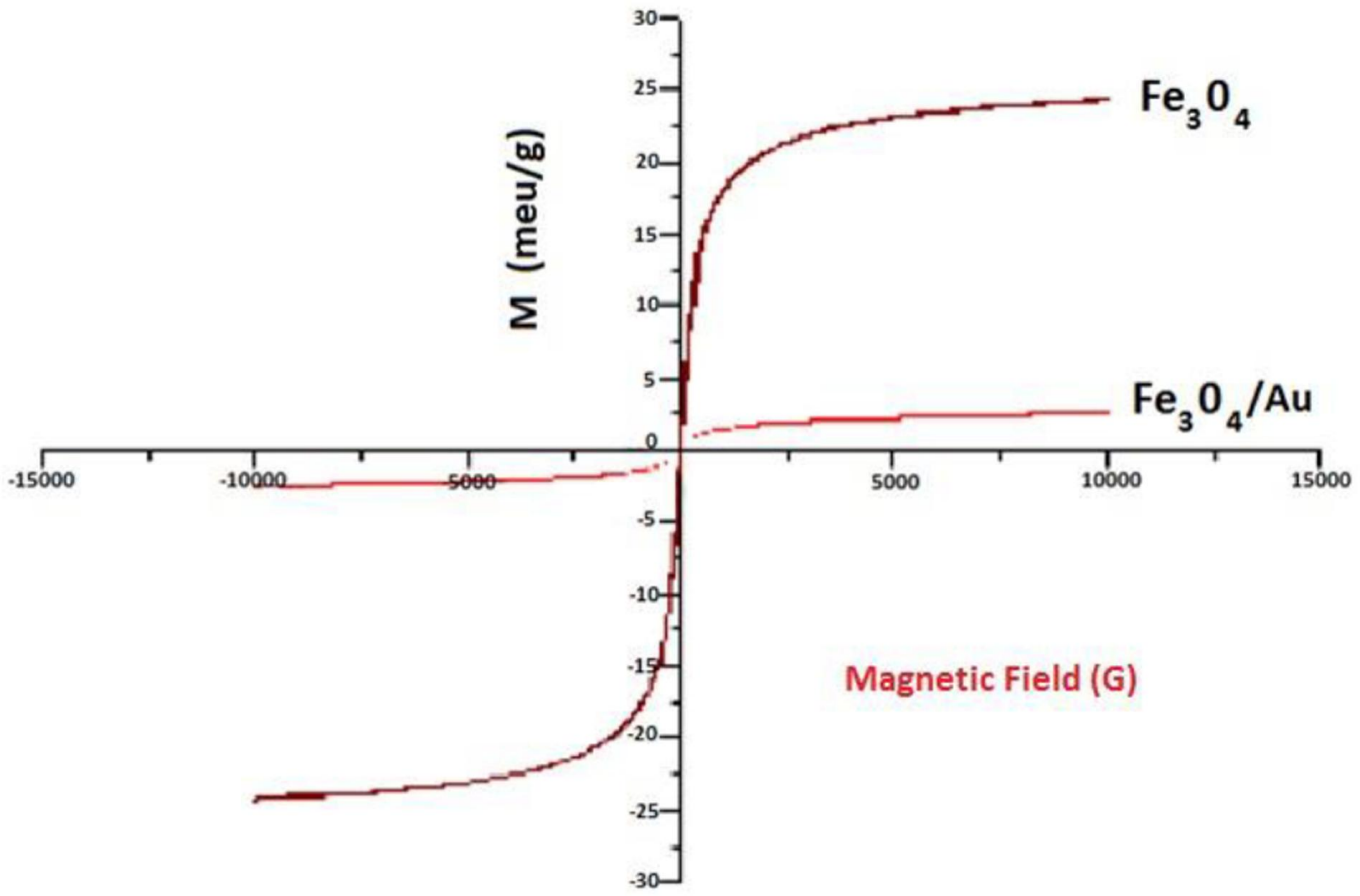
Vibrating sample magnetometer curve of magnetic and gold‐coated magnetic nanoparticles

As shown in Figure 2, magnetite nanoparticles and gold‐coated MNPs in a 10,000 G magnetic field show 24 emu/g (Ms) and 3 emu/g (Ms) saturation magnetization, respectively. The results showed that the magnetic properties of nanoparticles decreased when the MNPs were coated with gold.

### 3.4 X‐ray diffraction analysis

**FIGURE 3:**
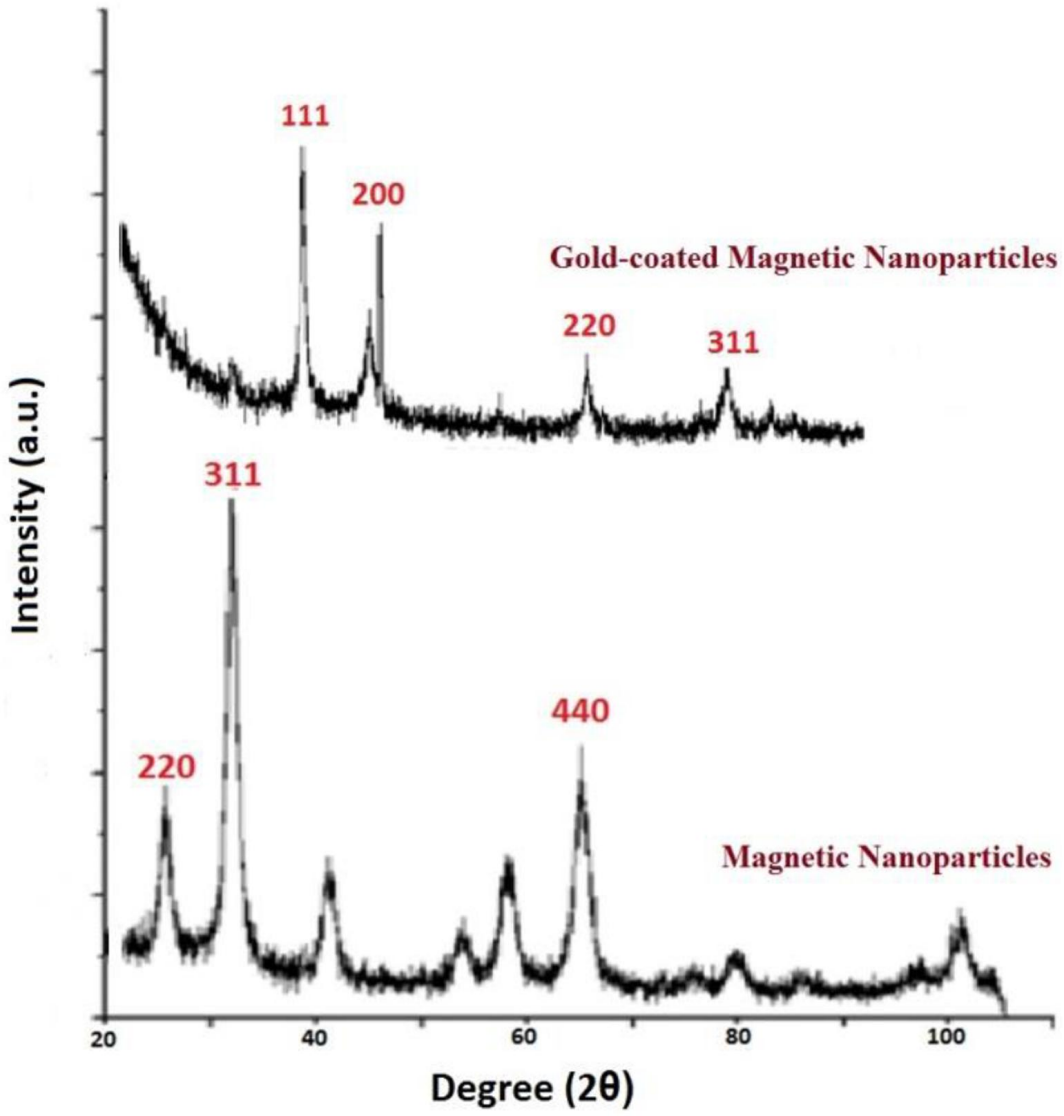
X‐ray diffraction pattern for magnetic nanoparticles and gold‐coated magnetic nanoparticles

To analyse the crystallographic structure of the magnetic nanoparticles and gold‐coated MNPs, XRD analysis was performed. The results are shown in Figure 3.

The peaks of MNPs and gold‐coated MNPs respectively correspond to the standard peaks of MNPs and gold‐coated superparamagnetic iron oxide nanoparticles (Au @ SPIONs) characteristic diffraction.

### 3.5 X‐ray photoelectron spectroscopy analysis

XPS is a quantitative analytical tool, a surface that is sensitive to the atomic composition of the outermost 10 nm of a sample surface. Therefore, XPS was used to analyse the chemical composition of gold‐coated MNPs. The binding energy for the C (1s) peak (284.6 eV) was used as an internal reference [39, 40].

**FIGURE 4:**
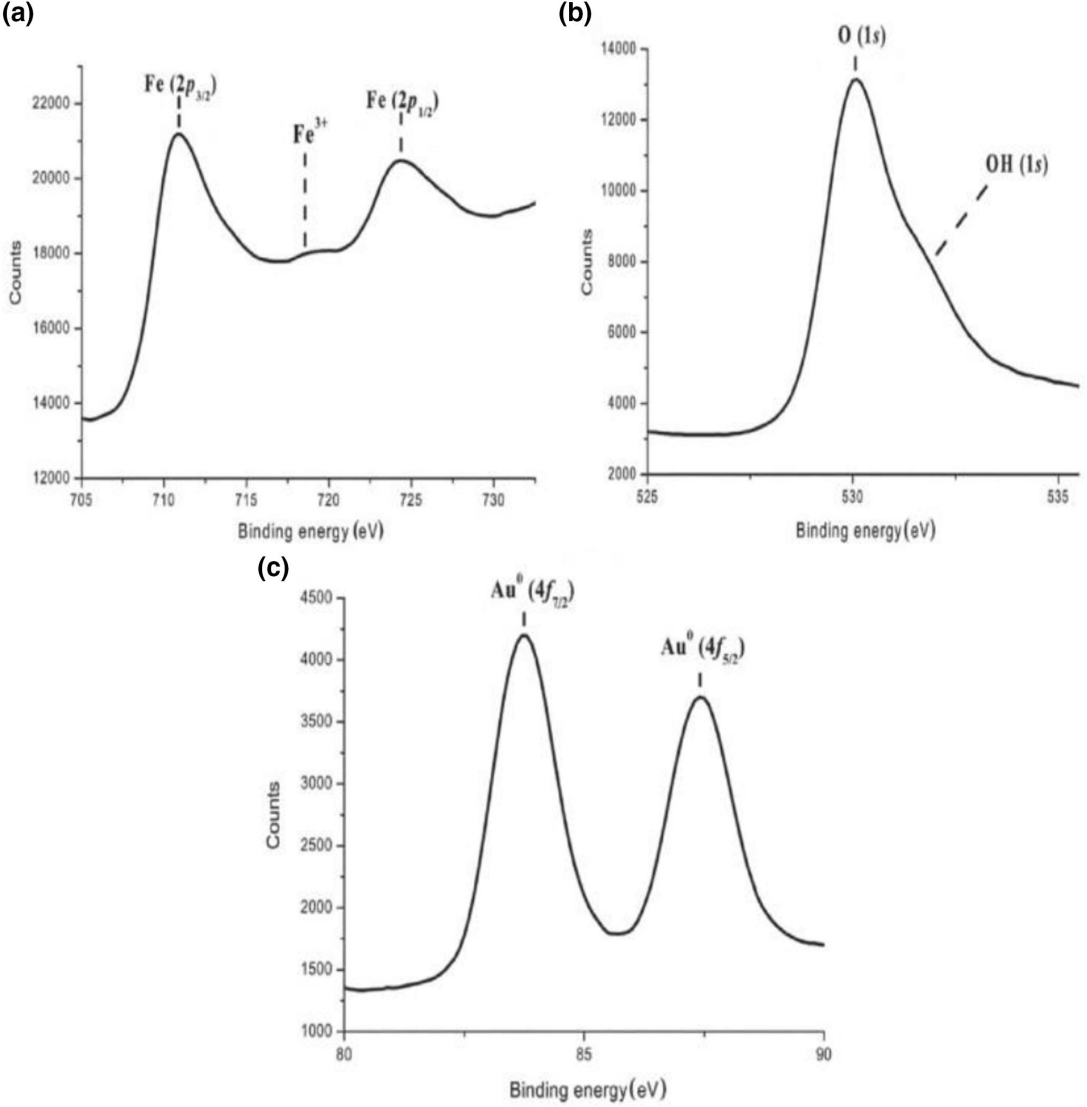
X‐ray photoelectron spectroscopy spectra of Au @ SPIONs nanoparticles. (a) Fe (2p), (b) O (1s), and (c) Au (4f). Au @ SPIONs, gold‐coated superparamagnetic iron oxide nanoparticles

Figure 4 shows the XPS spectra in different spectral regions corresponding to different elements for the gold‐coated MNPs. It displays the binding energies for Fe (2p3/2), Fe (2p1/2), O (1s), Au (4f7/2), and Au (4f5/2) of Au @ SPIONs nanoparticles. Figure 4(a) depicts two intense bands with binding energies of 710.8 and 724.0 eV, which are assigned to Fe (2p3/2) and Fe (2p1/2), respectively. Both bands consist of Fe^2+^ (of FeO) and Fe^3+^ (of Fe2O3) peaks and are typical characteristics of the Fe_3_O_4_ structure. The broad satellite peak at ∼ 718.5 eV corresponds to Fe^3+^. The Fe (2p3/2) peak shifted slightly from 711.2 to 710.8 eV owing to the addition of Au atoms [41, 42]. This indicates a strong electronic interaction between Au and Fe_3_O_4_. Figure 4(b) shows the XPS O (1s) spectrum of Au @ SPIONs nanoparticles. It consists of a major peak at 529.8 eV and a shoulder band at 531.5 eV, assigned to the O in Fe–O and O–H groups near iron, respectively [43]. The appearance of the hydroxyl group peak shows the surface adsorption of water molecules on Au @ SPIONs nanoparticles.

Figure 4(c) shows the XPS Au (4f) of Au @ SPIONs nanoparticles. Gold in the metallic state was detected with two broad intense bands: one at 83.6 eV for Au^0^ (4f7/2) and the other at 87.3 eV for Au^0^ (4f5/2) [44]. These results demonstrate the presence of Au^0^ atoms on the Au @ SPIONs nanoparticles. Moreover, the ratio of Fe to O was determined to be ∼1–1.3 according to the relative peak areas and their corresponding sensitivity factors. Thus, it well demonstrates the presence of Au^0^ atoms and Fe_3_O_4_ in the nanoparticles.

### 3.6 Transmission electron microscopy analysis

**FIGURE 5:**
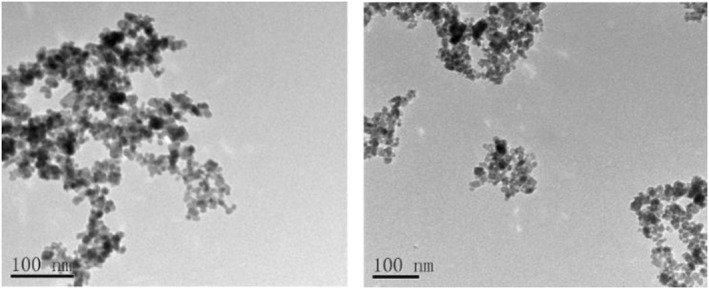
Transmission electron microscopy of magnetic nanoparticle

TEM was used to determine the true size distribution of the nanoparticles owing to limitations caused by the working principle of the zeta‐sized device. The TEM image of MNPs is blurry because the magnetic characteristics of the nanoparticles cause them to aggregate on the grid and interact with the particles of the electron beam. According to the results obtained from TEM images, the sizes of MNPs were around 10–15 NM (Figure 5).

**FIGURE 6:**
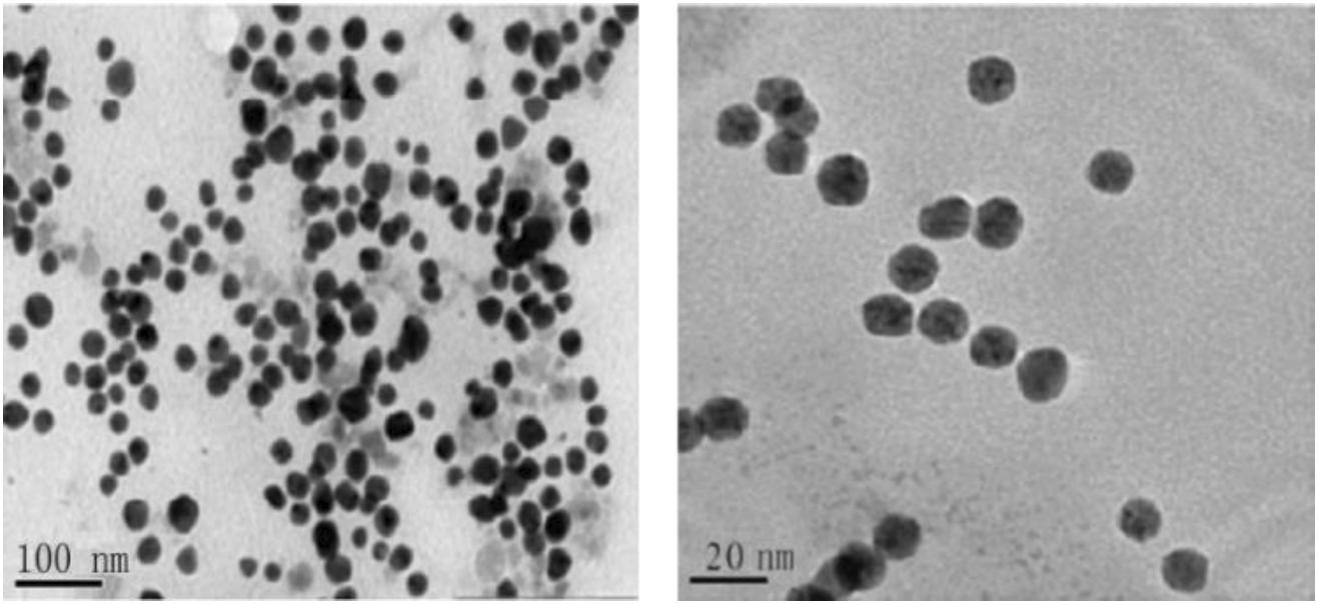
Transmission electron microscopy of gold‐coated magnetic nanoparticles

Figure 6 shows a TEM image of gold‐coated MNPs. Variations in the TEM image of MNPs is a reason for gold coating. According to the values obtained from the TEM image, 90% of nanoparticles are 20–30 NM.

### 3.7 Ninhydrin test

A ninhydrin test was used to show that antibodies bind to the modified nanoparticles. For this purpose, 500 μl of antibody‐immobilised gold‐coated nanoparticles was added to 500 μl of ninhydrin solution; after 4 min of holding in boiling water, the suspension turned black. The black colour is evidence that there is an antibody in the suspension.

### 3.8 Antibody‐nanoparticle binding rate

**FIGURE 7:**
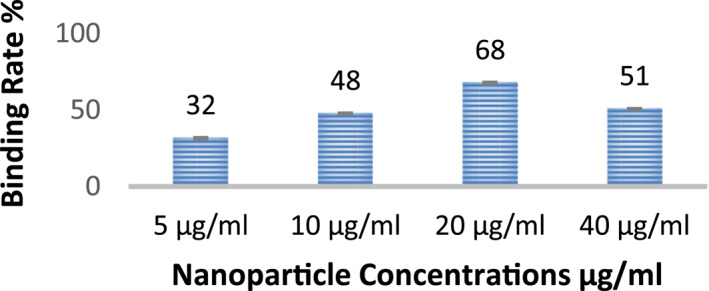
Antibody‐nanoparticle binding rate

In this experiment, four different nanoparticle concentrations (5, 10, 20, and 40 μg/ml) were treated with a constant amount of the antibody (10 μg/ml). After that, a Cobas Integra 400 Plus device was used to obtain an antibody binding percentage. The highest binding rate was for 20 μg/ml concentration of the nanoparticle (Table 2,  Figure 7)

**TABLE 2:** Binding quantity and rates of antibody and nanoparticles

Monoclonal antibody (µg/ml)	Np (µg/ml)	Binding quantity (µg)	Binding rate (%)	STDEV.S
10	5	3.2	32	0.16
10	10	4.8	48	0.2
10	20	6.8	68	0.34
10	40	5.1	51	0.1

Abbreviation: STD, standard deviation.

### 3.9 Cytotoxicity assay

**FIGURE 8:**
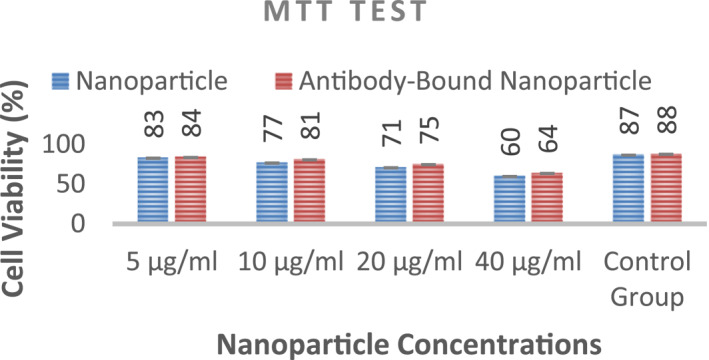
Effects of nanoparticle concentration on cell viability to determine toxicity limit

The MTT (3‐(4,5‐dimethylthiazol‐2‐yl)‐2,5‐diphenyl tetrazolium bromide) test was used to determine the toxicity of nanoparticles using the 3T3 cell line (standard fibroblast cell line). Nanoparticle solution was prepared at four different concentrations (5, 10, 20, and 40 μg/ml) (Table.3, Figure 8).

**TABLE 3:** Cell viability

	5 μg/ml	10 μg/ml	20 μg/ml	40 μg/ml	Control group
Nanoparticle	83%	77%	71%	60%	87%
Antibody/Nanoparticles	84%	81%	75%	64%	88%

Cell viability values in the cytotoxicity test can be accepted for 5, 10, and 20 μg/ml of nanoparticles. According to the results of the antibody‐nanoparticle binding rate and MTT test, a concentration of 20 μg/ml was used for nanoparticles in the experiments.

### 3.10 Radio‐frequency hyperthermia method

Heat generated using RF waves in the presence of synthesised nanoparticles was studied in our team's previous work [43, 44]. RF (144.00015 MHz, 120 W) exposed to nanoparticles revealed that the highest heat rate record belonged to the MNPs compared with different nanostructures captured by the infrared thermal camera (FLIR i5).

**FIGURE 9:**
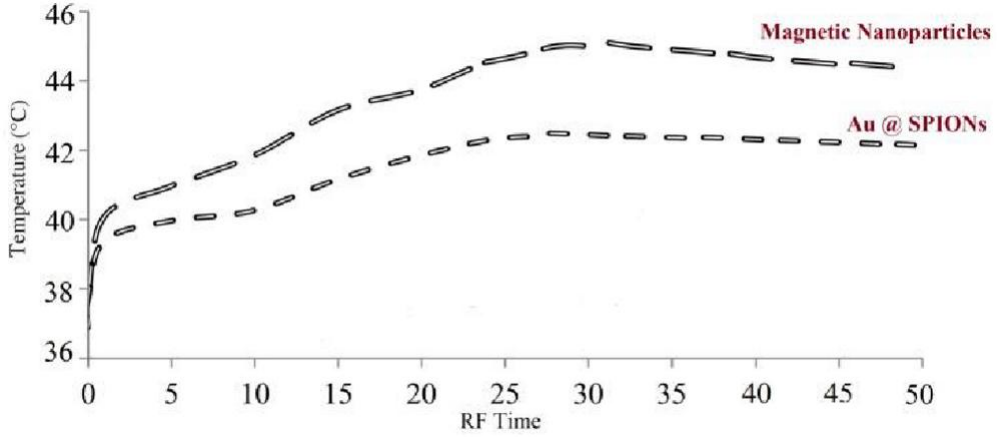
Difference in heat production in nanoparticles dispersion owing to radio‐frequency exposure

In Figure 9, the dashed line represents Au @ SPIONs (36–42.3°C), and the long dashed line denotes MNPs with the highest heat generation rate (36–44.6°C) in the presence of RF power.

### 3.11 In vitro studies

In the in vitro study, antibody‐bound nanoparticles interacted with MCF‐7 cells. The antibody‐bound nanoparticles were added to the MCF‐7 cells at a concentration of 20 μg/ml in two groups, and the RF generator was operated in one group at 120 W output power for 50. All groups were incubated for 48 h and then cells were stained with the dual staining apoptosis‐necrosis method. Then, images were taken using a DAPI filter on a fluorescence microscope (Figures 10.1 and 10.2).

**FIGURE 10:**
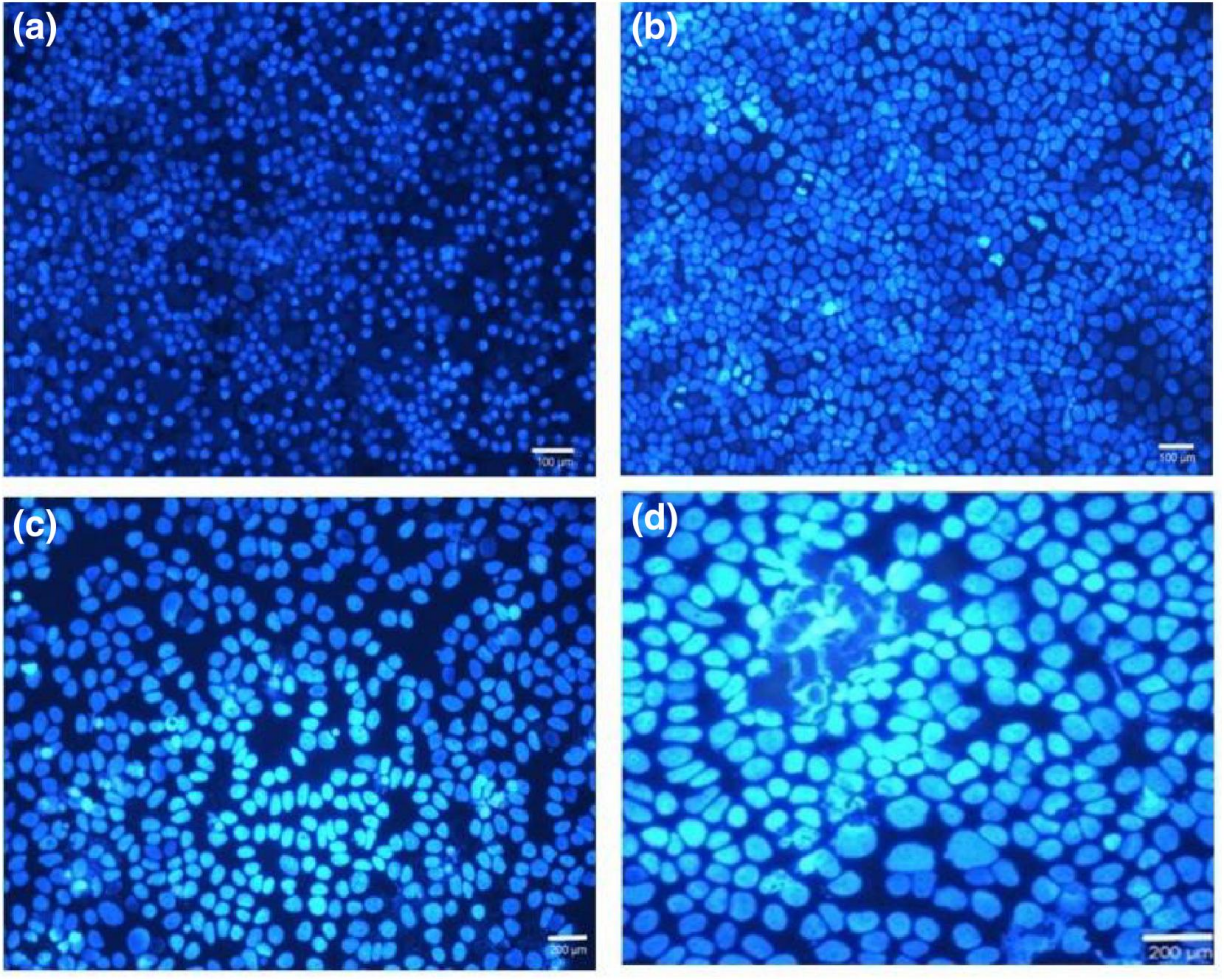
MCF‐7 cells were analysed by DAPI (4′,6‐diamidino‐2‐phenylindole) filtration (a). The interaction of Au @ SPIONs (20 μg/ml) with MCF‐7 cells was analysed by DAPI filtration (b). The interaction of antibody‐bound Au @ SPIONs (20 μg/ml) with MCF‐7 cell before radio‐frequency (RF) was analysed by DAPI filtration (c). The interaction of antibody‐bound Au @ SPIONs (20 μg/ml) with MCF‐7 cell after RF was analysed by DAPI filtration (d). Au @ SPIONs, gold‐coated superparamagnetic iron oxide nanoparticles

**FIGURE 10:**
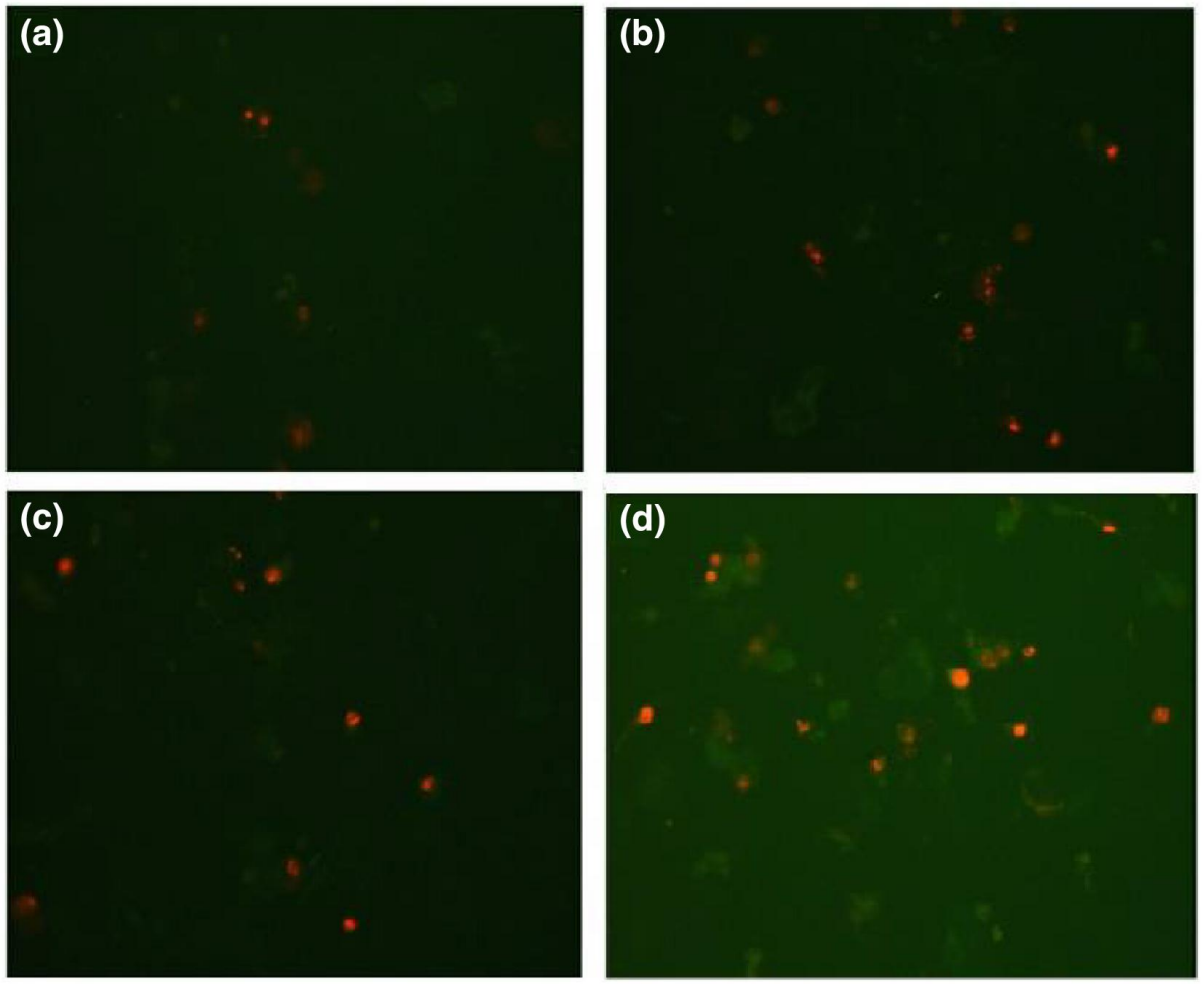
MCF‐7 cells were analysed by fluorescein isothiocyanate (FITC) (480–520 nm wavelength) (a). The interaction of Au @ SPIONs (20 μg/ml) with MCF‐7 cells was analysed by FITC (b). The interaction of antibody‐bound Au @ SPIONs (20 μg/ml) with MCF‐7 cell before radio‐frequency (RF) was analysed by FITC (c). The interaction of antibody‐bound Au @ SPIONs (20 μg/ml) with MCF‐7 cell after RF was analysed by FITC (d). Au @ SPIONs, gold‐coated superparamagnetic iron oxide nanoparticles

**FIGURE 11:**
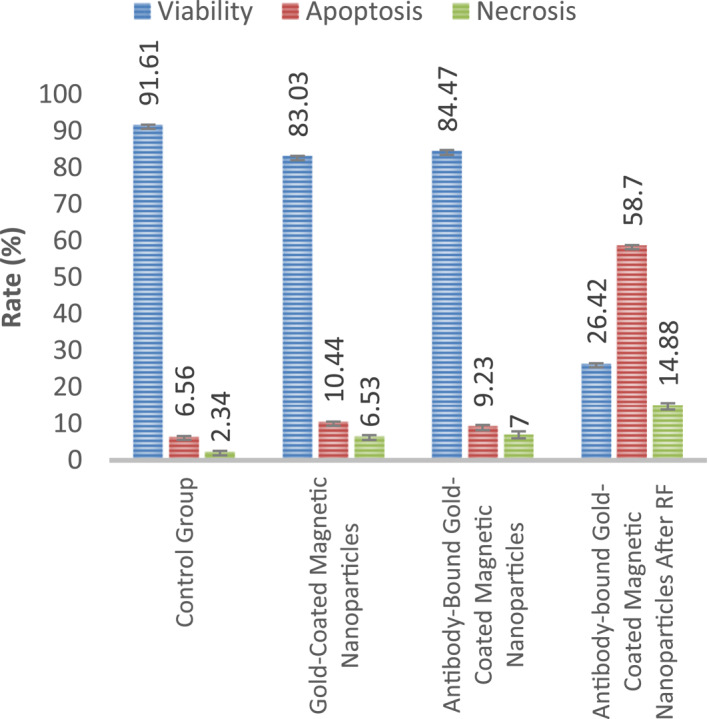
Results from cell culture experiments

Results obtained from cell culture experiments (Figure 11) show that hyperthermia successfully induces apoptosis and necrosis.

### 3.12 In vivo experiments (animal model studies)

In nude mice, a tumour formed with the injection of MCF‐7 cell solution. Mice were divided into three groups according to the injection of nanoparticles during the experiment (control, intratumour injection, and intravenous injection groups).

**FIGURE 12:**
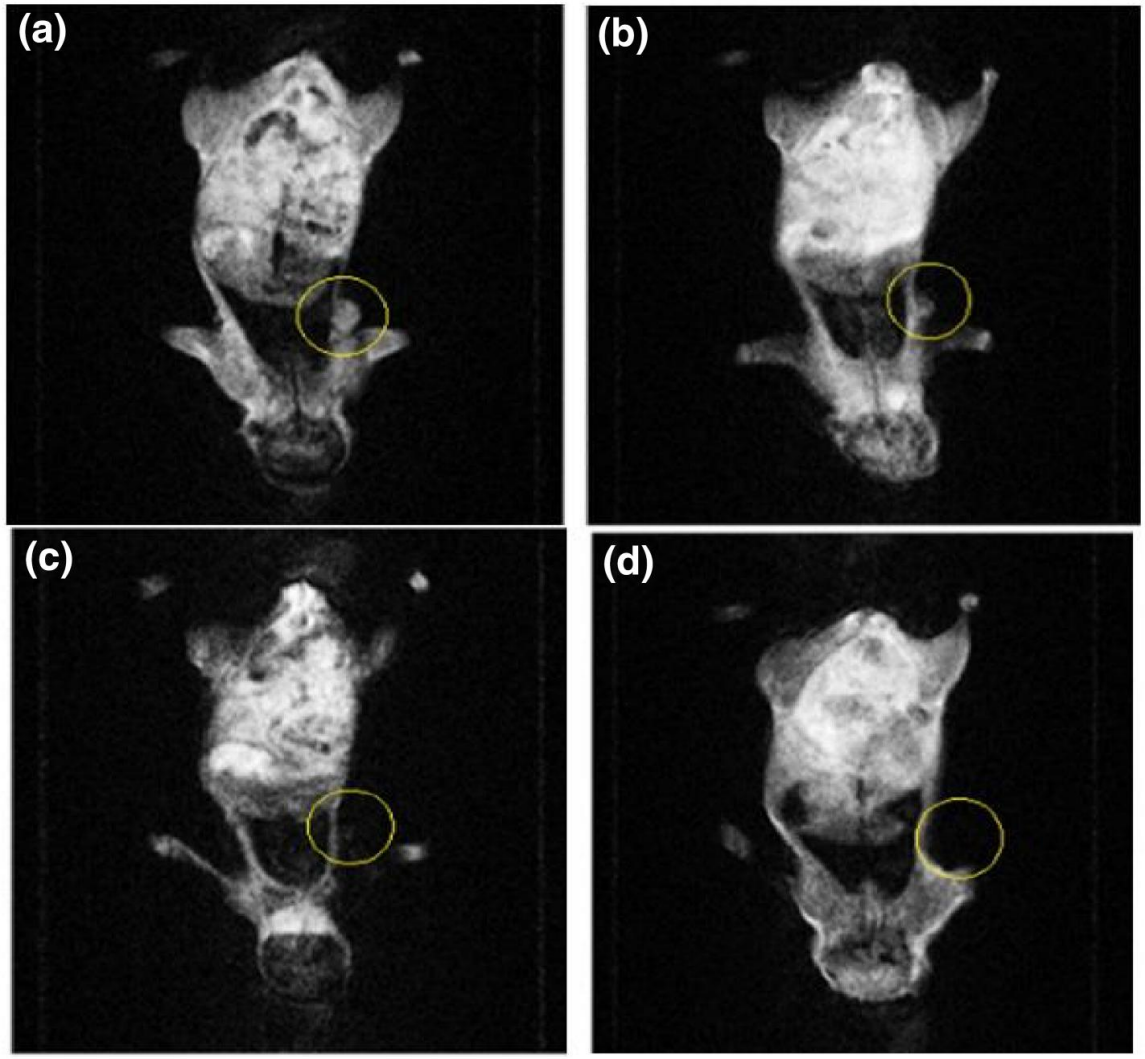
Magnetic resonance imaging of intravenous injection after (a) 0 h, (b) 2 h, (c) 4 h, and (d) 24 h

**FIGURE 13:**
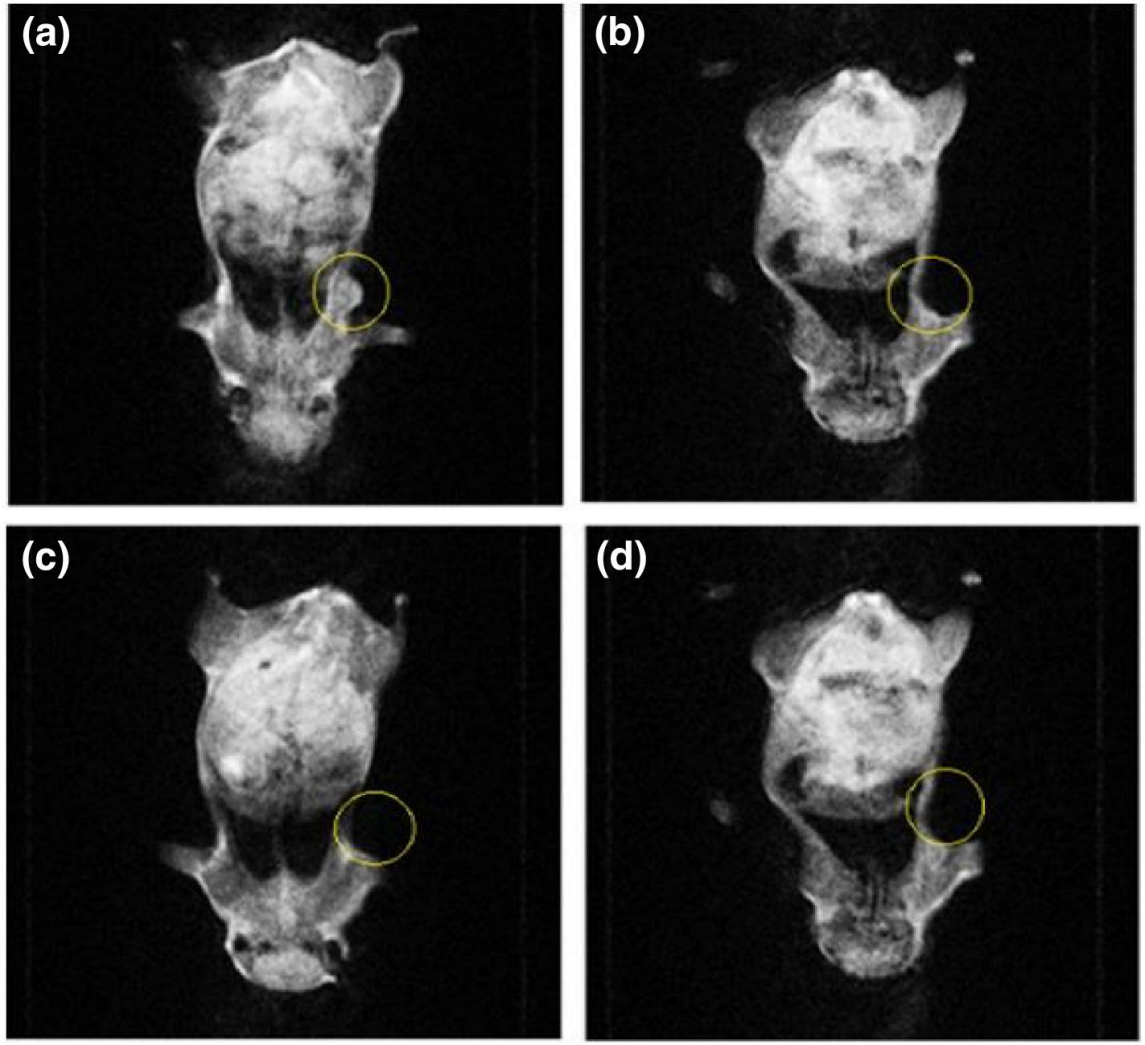
Magnetic resonance imaging of intravenous injection after (a) 0 h, (b) 2 h, (c) 4 h, and (d) 24 h

Nanoparticles (50 μl) were injected into the tumour for the intratumour group and 150 μl of nanoparticles into the tail venously for the intravenous group. MRI was used to show the fate of nanoparticles in the body. Because of the magnetic properties of the injected nanoparticles, the MRI image of this part becomes black when the nanoparticles are collected from any part of the body (Figures 12 and 13).

As shown in Figure 12, nanoparticles begin to bind to tumour cells 2 h after intravenous injection; after 4 h, nanoparticles are completely attached to tumour cells. The disappearance of the tumour means the collection of nanoparticles in the tumour. After 24 h, the tumour looks black owing to the binding of nanoparticles to tumour cells.

As shown in Figure 13, nanoparticles begin to bind to tumour cells after intratumour injection. Antibodies immobilised to nanoparticles are specific antibodies to MCF7 cells that attach to receptors on the surface of tumour cells. Thus, gold‐coated MNPs accumulate in tumours; therefore, the tumour looks black.

The results obtained from MRI images show that the designed nanoparticles bind successfully to cancerous cells.

### 3.13 Application of radio‐frequency

**FIGURE 14:**
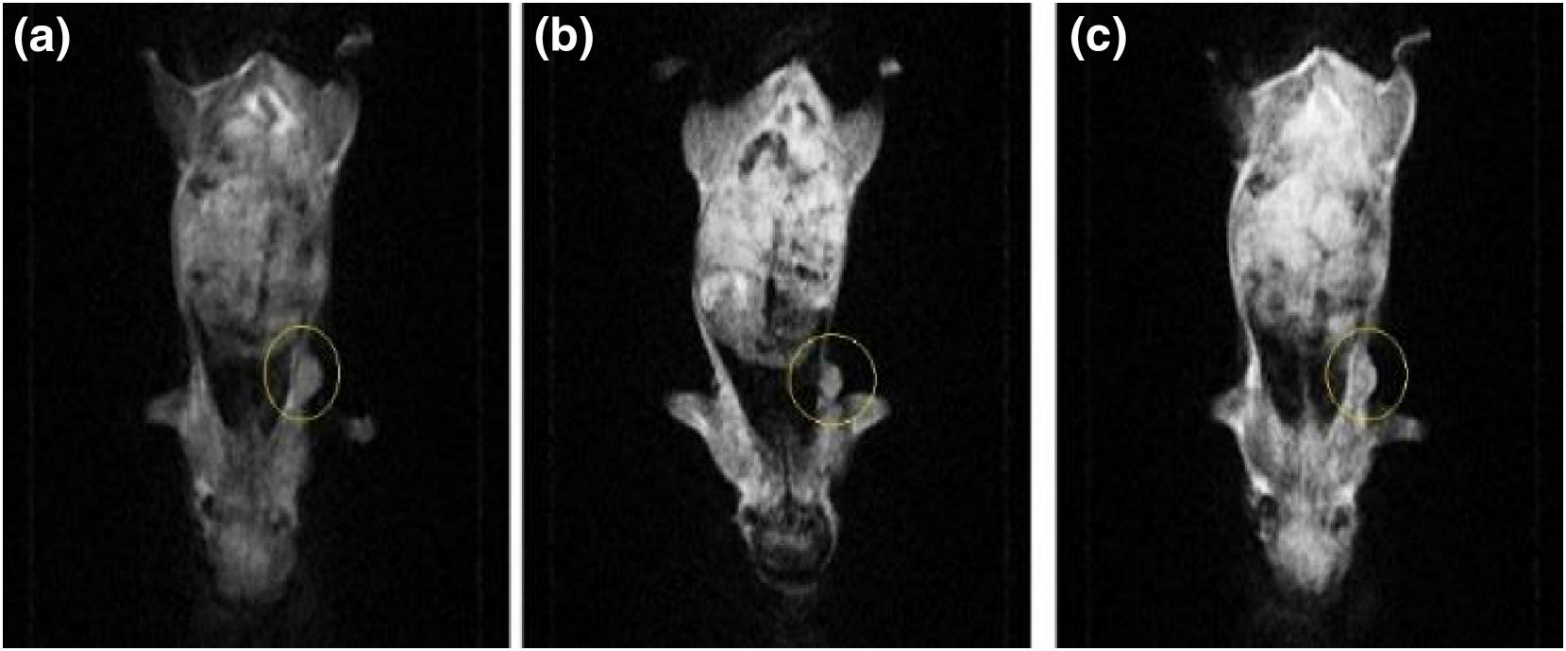
Magnetic resonance imaging after radio‐frequency application in intravenous injection group after (a) 1 week; (b) 2 weeks; (c) 3 weeks

**FIGURE 15:**
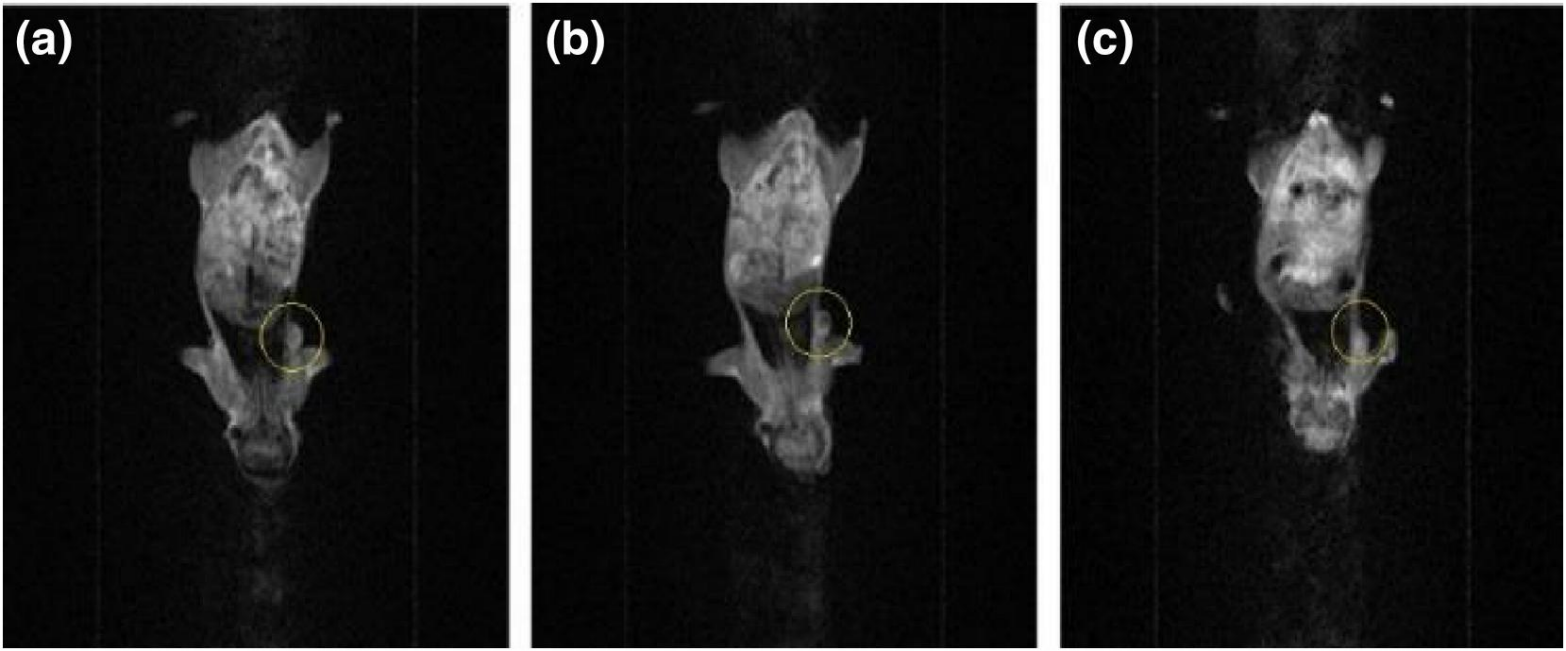
Magnetic resonance imaging after radio‐frequency application in intratumour injection group after (a) 1 week; (b) 2 weeks; (c) 3 weeks

The nude mice were weighed one by one, and then anaesthesia was performed by inhalation using isoflurane. RF application on animals was performed once (120 W/144.00,015 MHz/50 min) in the intravenous injection and intratumour injection groups (Figures 14 and 15). We selected these data because of results from our previous studies [45, 46]. The increase in temperature in the tumour area was controlled by a thermal thermometer during the application of RF.

**FIGURE 16:**
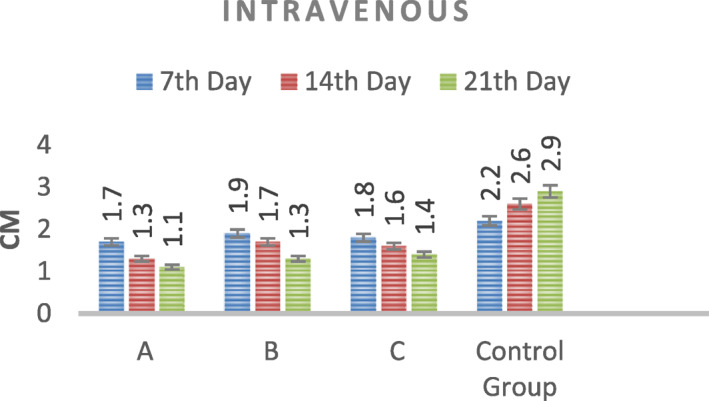
Data of intravenous injection group after radio‐frequency application

According to the results, when mice were administered RF after intravenous injection, an approximately 34% reduction in tumour size was recorded at the end of the third week (Figure 16).

**FIGURE 17:**
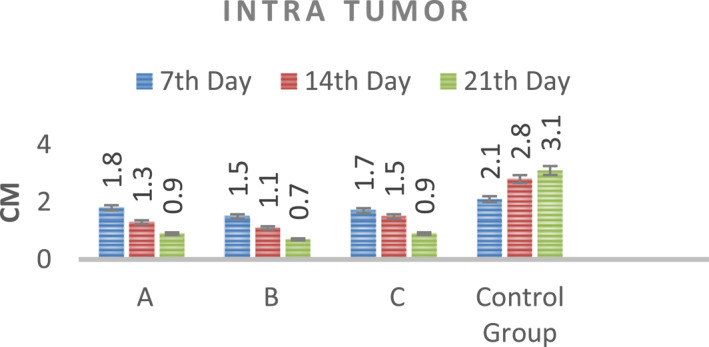
Data of intratumour injection group after radio‐frequency (RF) hyperthermia

An approximately 53% reduction in tumour size was noted at the end of the third week after RF application in the tumour injection group. A reduction in tumour size as a result of RF hyperthermia occurs owing to apoptosis and necrosis of cancer cells (Figure 17).

**FIGURE 18:**
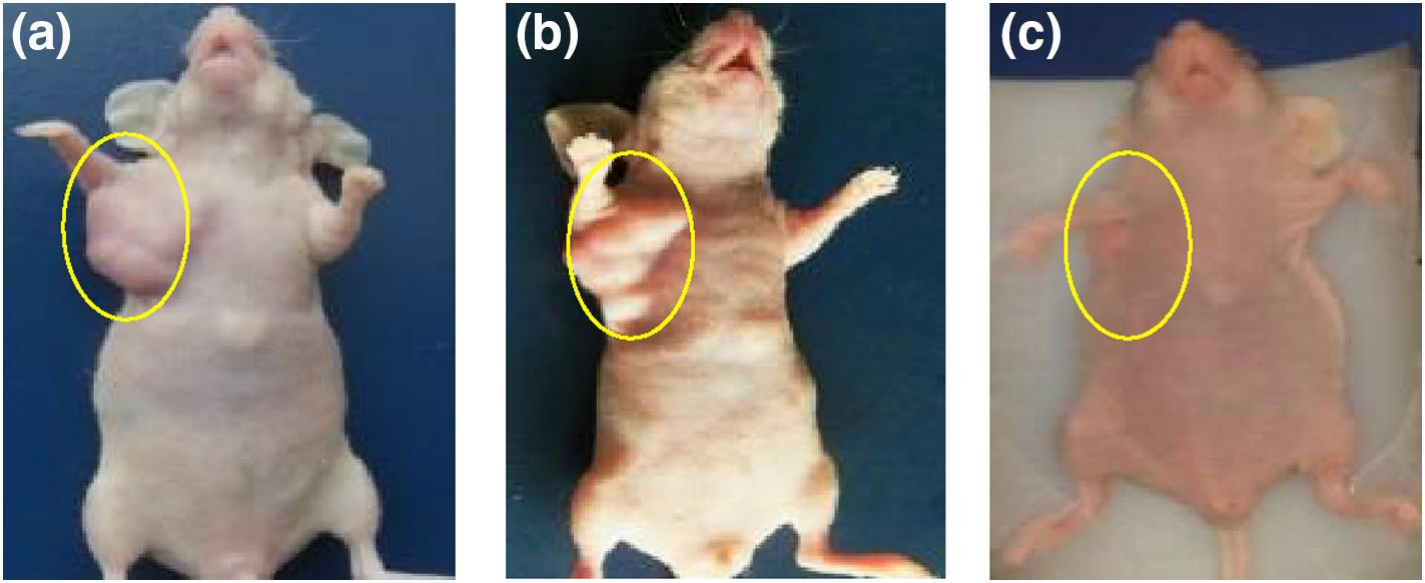
Local photo from mouse: (a) immediately after radio‐frequency (RF) application; (b) 1 week and (c) 3 weeks after RF application in intratumour injection group

**FIGURE 19:**
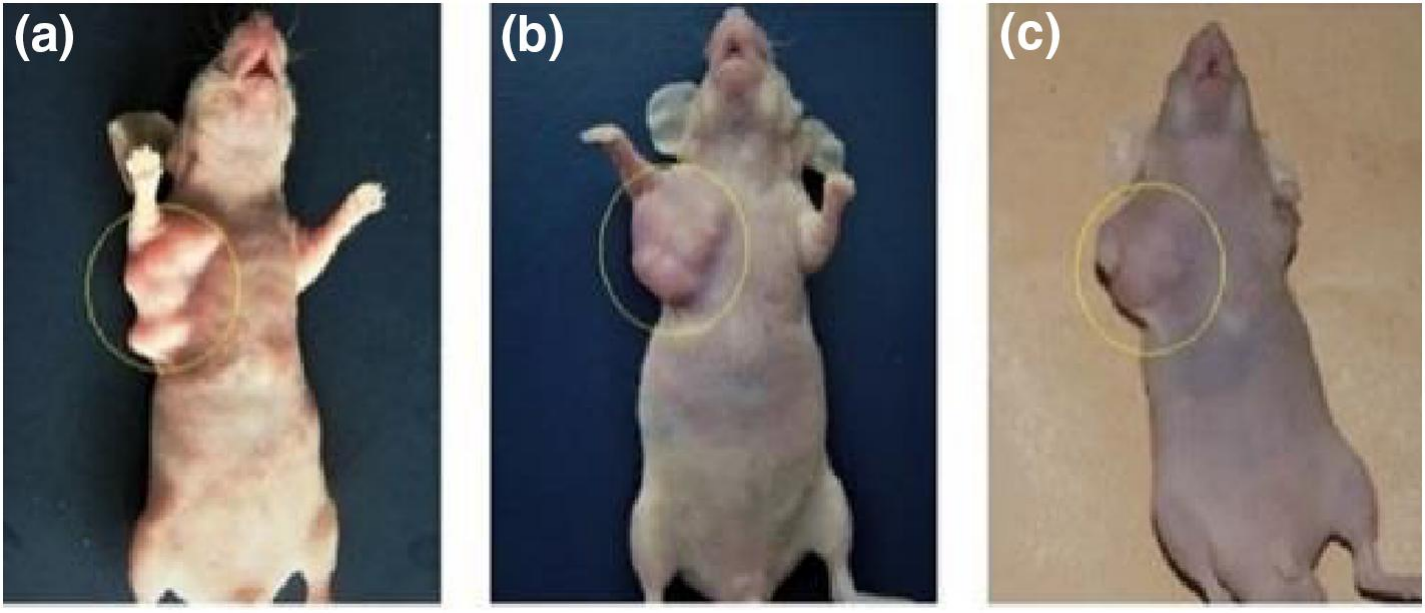
Local photo from mouse: (a) immediately after nanoparticle injection; (b) 1 week and (c) 3 weeks after nanoparticle injection in control group

The data of experiments show that hyperthermia with RF (by gold‐coated magnetic nanoparticles) causes a significant loss of tumour cells within 3 weeks (Figures 18 and 19)

**FIGURE 20:**
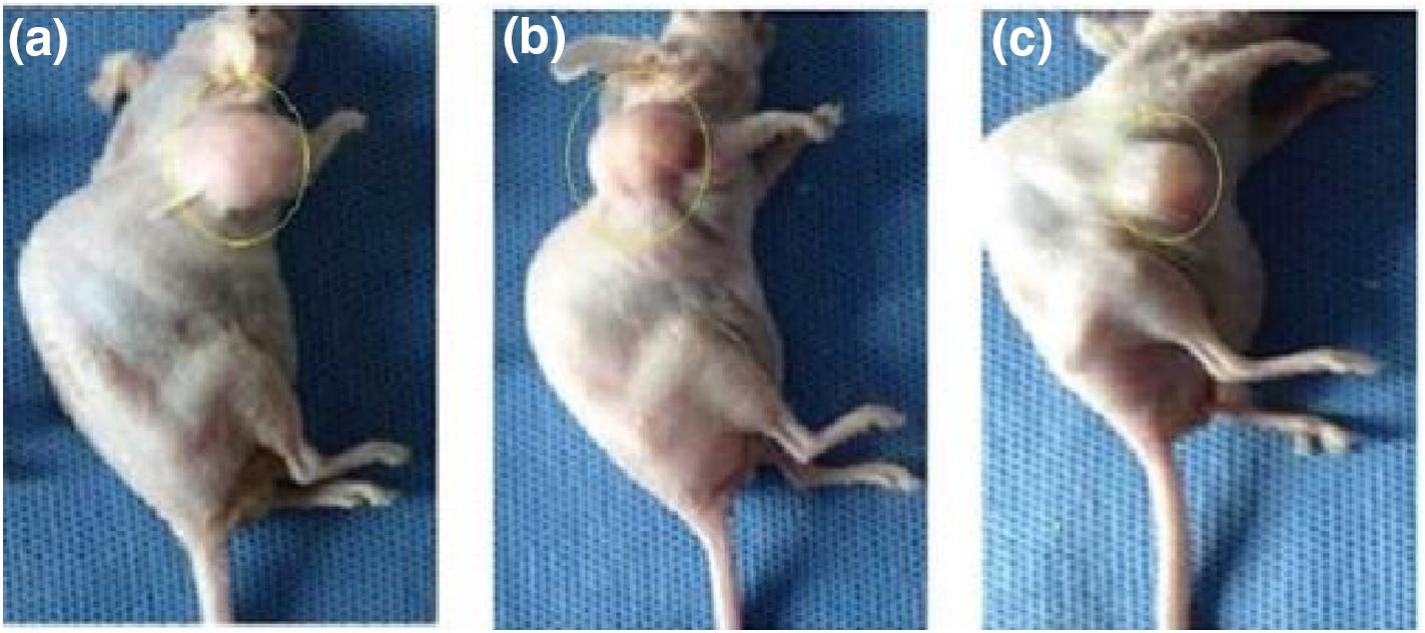
(a) immediately after radio‐frequency (RF) application; (b) 1 week and (c) 3 weeks after RF application in intravenous injection group

**FIGURE 21:**
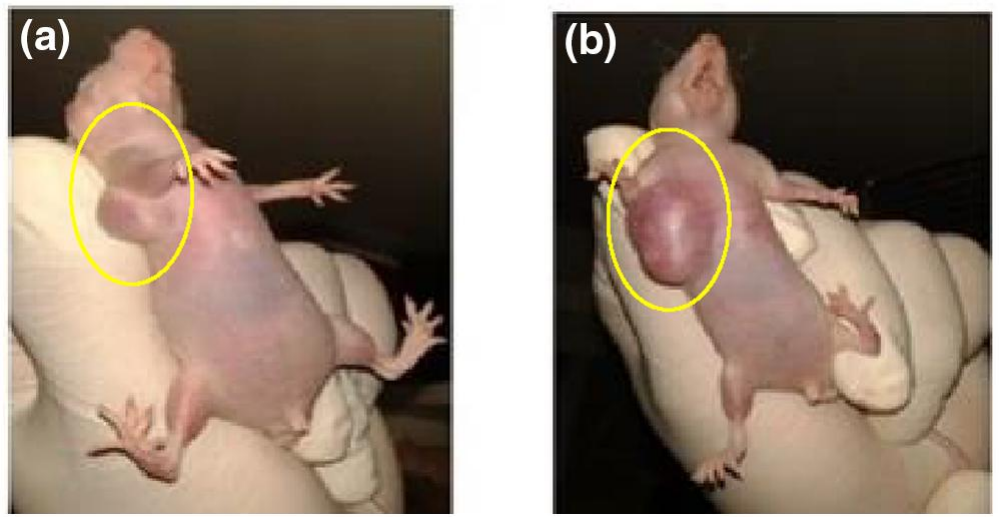
(a) control group immediately after nanoparticle injection; and (b) 3 weeks after nanoparticle injection

According to the data obtained from the experiments on mice, the rates of apoptosis and necrosis increased in cancerous cells when nanoparticles were injected into the tumour. Thus the tumour size in the intratumour injection group is smaller than that in the intravenous injection group (Figures 20 and 21)

The difference in the tumour size between the intratumour and intravenous groups is due to the different concentrations of nanoparticles in the tumour. The nanoparticle concentration decreases when it is injected intravenously. Because the nanoparticle circulates in all organs of the body, a small amount of nanoparticles is collected in the tumour.

### 3.14 Histopathology evaluation of tumours after radio‐frequency application (treatment)

**FIGURE 22:**
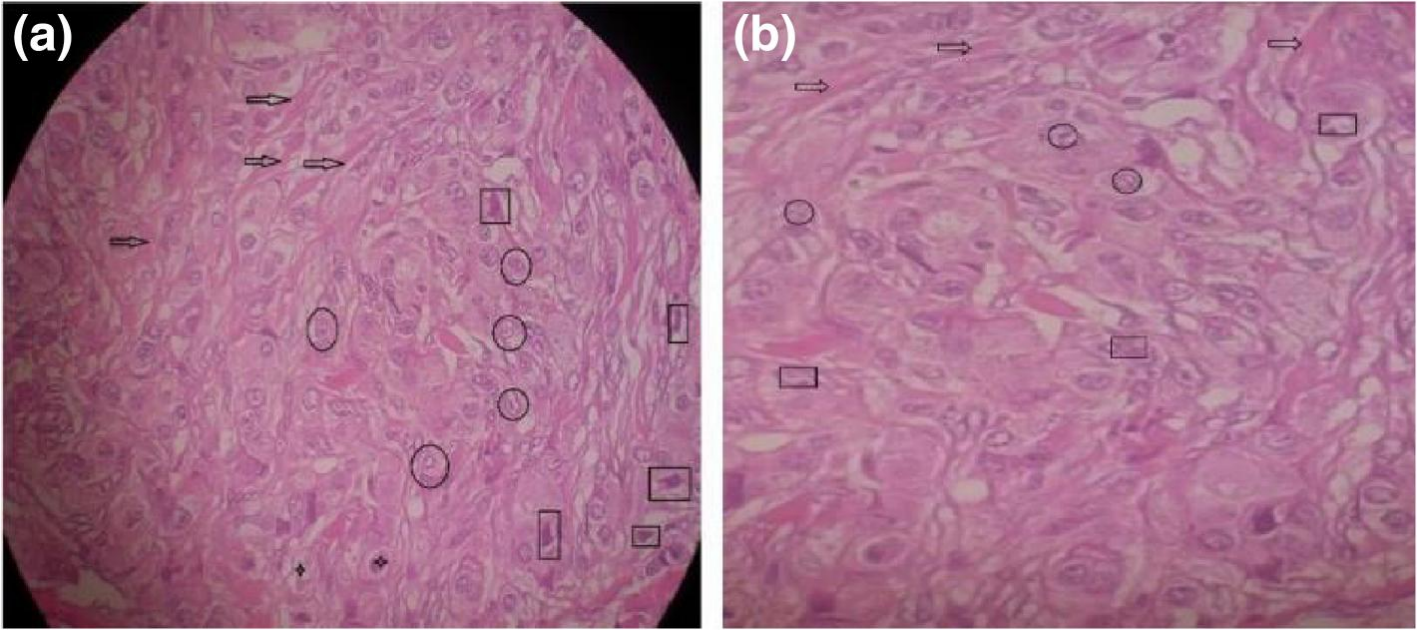
Histopathology evaluation of control group (a) → desmoplasia, ○ tumour cells, □ malignant tumour cells, □ macrophage cells; (b) ○ mitosis (mitotic division)

Tumour histopathology was evaluated in all groups after treatment. The accumulation of collagen fibres in the tissue is seen in the control group. Connective tissue is formed instead of normal tissue (desmoplasia). Tumour cells are seen with torn nuclei intensely and irregularly in all tissues. Macrophage cells and mitotic divisions are seen in tumour tissue. These are positive indices for tumour detection (Figure 22).

**FIGURE 23:**
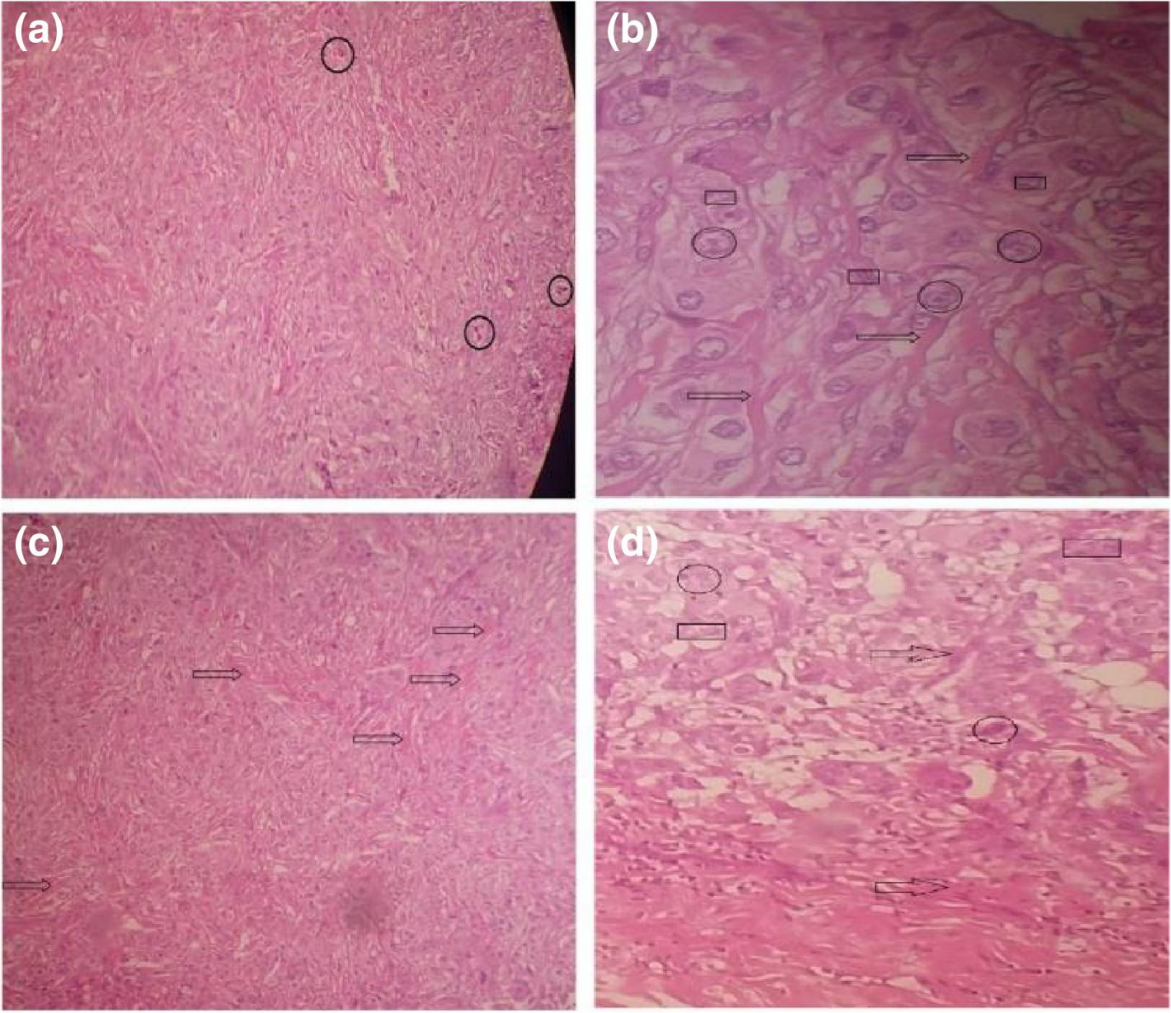
Histopathological microscopic images of tumours after radio‐frequency hyperthermia treatment: (a, b) intratumour group; (c, d) intravenous group). ○, tumour Cells; □, histiocytes; →, fibrotic tissues

The number of tumour cells decreases in the treated groups and the remains of tumour cells are observed in some places. The cancerous cells were deformed and their morphology changed after treatment. Histiocytes are responsible for removing cells undergoing apoptosis and necrosis after treatment. There were fibrotic tissues in histopathology images. The formation of fibrous tissue shows that the tissue repairs the arrival of connective tissue and starts the repair period (Figure 23).

## 4 CONCLUSION

Much evidence was found confirming the damaging effect of nanoparticle‐mediated RF hyperthermia on morphological changes in tumour tissues. Investigation of the heat generation of nanoparticles induced by RF revealed that the highest temperature was produced by Au @ SPIONs compared with other nanoparticles. We observed 10.44% ± 0.89% (Au @ SPIONs) and 9.23% ± 1.43% (antibody‐bound Au @ SPIONs) apoptosis when nanoparticles were added to MCF‐7 cells. After the application of RF hyperthermia, 58.7% ± 1.12% apoptosis and 14.8% ± 1.63% necrosis were calculated in MCF‐7 cells. According to the results, after RF hyperthermia was applied, a significantly high number of the cells underwent apoptosis. Consequently, nanoparticles were designed and synthesised, and the established RF system was successful.

According to the results of in vivo studies, the tumour image is black on MRI after 24 h because there is adhesion of nanoparticles to cancer cells after intratumour injection. In the intravenous group, nanoparticles begin to bind to tumour cells 2 h after injection. After 4 h, the nanoparticles are bound to all tumour cells so that the tumour image becomes black. An approximately 34% reduction in the tumour size was noted at the third week when RF hyperthermia was applied after intravenous injection of mice. At the end of the third week, about 53% reduction in tumour size was recorded, when RF hyperthermia was applied after the intratumour injection. The reduction in tumour size as a result of RF hyperthermia is the result of the apoptosis of cancer cells. There is a difference between the intratumour and intravenous groups (the rate of reduction in the tumour size) because the nanoparticle in the intravenous group is less collected than in the intratumour injection group. Also, pathology and histology studies proved that RF exposure can penetrate fibrotic tissue, which is the main component of tumour tissue. Histological necrosis in the tumour tissue proves the proper diffusion of antenna modulus RF waves creating histological damage in the tissue.

As a result, the most important originality of this work is that antibody‐bound gold‐coated MNPs (designed and synthesised here) were successfully connected to tumour cells, and tumour size decreased after RF hyperthermia owing to apoptosis and necrosis of tumour cells.
